# Astragalus Polysaccharide Suppresses the Expression of Adhesion Molecules through the Regulation of the p38 MAPK Signaling Pathway in Human Cardiac Microvascular Endothelial Cells after Ischemia-Reperfusion Injury

**DOI:** 10.1155/2013/280493

**Published:** 2013-11-05

**Authors:** Zhu Hai-Yan, Gao Yong-Hong, Wang Zhi-Yao, Xu Bing, Wu Ai-Ming, Xing Yan-Wei, Liu Bei, Lou Li-Xia, Chen Li-Xin

**Affiliations:** ^1^Institute for Cardiovascular Disease, Dongzhimen Hospital Affiliated to Beijing University of Chinese Medicine, Beijing 100700, China; ^2^Key Laboratory of Chinese Internal Medicine of Ministry of Education and Beijing, Dongzhimen Hospital Affiliated to Beijing University of Chinese Medicine, Beijing 100700, China; ^3^Eye Hospital, China Academy of Chinese Medical Sciences, Beijing 100040, China; ^4^Beijing Tibetan and Ethnic Medicine Hospital, 100053, China; ^5^Guang'anmen Hospital, China Academy of Chinese Medical Sciences, Beijing 100053, China; ^6^World Federation of Chinese Medicine Societies, Beijing 100029, China

## Abstract

Astragalus polysaccharide is a major component of radix astragali, a vital qi-reinforcing herb medicine with favorable immune-regulating effects. In a previous animal experiment, we demonstrated that astragalus polysaccharide effectively alleviates ischemia-reperfusion injury (IRI) of cardiac muscle through the regulation of the inflammatory reactions. However, the relationship between this herb and the cohesion molecules on the cell surface remains controversial. In this study, human cardiac microvascular endothelial cells (HCMECs) were used to validate the protective effects of astragalus under an IRI scheme simulated through hypoxia/reoxygenation in vitro. The results indicated that astragalus polysaccharide inhibited the cohesion between HCMECs and polymorphonuclear leukocyte (PMN) during IRI through the downregulation of p38 MAPK signaling and the reduction of cohesive molecule expression in HCMECs.

## 1. Introduction

Myocardial ischemia-reperfusion represents a milestone in the management of acute myocardial infarction; however, the management of ischemia-reperfusion injury (IRI) presents a medical conundrum. In IRI, the coronary artery is typically revascularised after reperfusion, although in more severe hemorrhagic infarctions, as observed in cardiac muscles supplied through the artery, the circulation of the distal ischemic area is blocked, resulting in serious microvascular damage. 

Until recently, the mechanisms underlying cardiac IRI have not been fully understood. It has been suggested that IRI results from a specific inflammatory response [1], where leukocyte participation is a pivotal step leading to injury and the microvascular endothelium is a key target. The molecular basis of IRI involves the interaction between vascular endothelial cells (VECs) and adhesion molecules on the surface of leukocytes and the regulation of adhesion molecule expression through associated factors [2, 3].

A recent study showed that the regulation of the signal transduction network is vital to regulate adhesion molecule expression [4]. The transcription of adhesion molecule requires the involvement of the mitogen-activated protein kinase (MAPK) signalling pathway and NF-*κ*B transcription factors [5, 6]. Notably, there is a close association between p38 MAPK and the injury in VECs [7]. Hypoxia, oxygen radicals, and other stresses stimulate VECs and activate the p38 MAPK-mediated secretion of adhesion molecules. The suppression of the associated signalling pathways significantly alleviates the prognosis of IRI [8].

There are some similarities between Chinese medicine and modern medicine concerning the comprehension of ischemic heart diseases [9]. Specifically, traditional Chinese medicine considers ischemias an obstruction of the heart meridian, contributing to poor blood flow, and the heart meridian is the counterpart of cardiac microvessel. Traditional Chinese medicine considers blood stasis and collateral obstruction as consequences of ischemia, whereas yang and qi deficiencies are the causes of this disease. Consequently, qi-tonifying and blood-activating herbs are commonly used to treat this medical condition. Astragalus is an important qi-tonifying herbal medicine whose unique effect has been fully demonstrated in clinical practice [10]. Astragalus polysaccharide is one of the main effective ingredients of this herb. In a previous study, we showed that astragalus polysaccharide exhibits superior anticardiac IRI action in animals. Specifically, this compound restored cardiac mechanisms, such as coronary blood flow, LPO content, superoxide dismutase activity, and so forth, to favourable levels after reperfusion [11, 12]. However, the influence of astragalus polysaccharide on the expression of adhesion molecules in VECs remains unknown. While some studies have indicated that this compound upregulates the expression of adhesion molecules [13], other studies suggest that astragalus polysaccharide downregulates adhesion molecule expression [14].

To avoid confusion through heterogeneity in cells, organs, and tissues, primary human cardiac microvascular endothelial cells were used as target cells, and *in vitro* hypoxia-reoxygenation was adopted to simulate ischemia-reperfusion under these conditions. The effects of astragalus polysaccharide on the cohesion of HCMECs and PMN during IRI, the expression of major adhesion molecules, and the regulation of signalling pathways were observed. 

## 2. Materials and Methods

### 2.1. Materials

Human cardiac microvascular endothelial cells (HCMECs), endothelial cell media, and cell digestion enzymes were purchased from the ScienCell Corporation, USA. Poly-l-lysine and SB203580 (p38 MAPK inhibitor) were obtained from Sigma, USA. Rose Bengal sodium salt was purchased from the Beijing Chemical Reagents Company. Astragalus polysaccharide (batch no.: Z20040086) was obtained from the Tianjin Cinorch Pharmaceutical Co., Ltd., China. Polymorphonuclear leukocyte (PMN) separation medium was obtained from the Institute of Hematology and Blood Diseases Hospital Chinese Academy of Medical Sciences. The SV Total RNA Isolation System and Reverse Transcription System were purchased from the Promega Corporation, USA. Power SYBR Green kits were purchased from ABI. A mouse monoclonal antibody against E-selectin, (product no.: SC-137054), goat polyclonal antibody against P-selectin (product no.: SC-6941), rabbit polyclonal antibody against p38*α*/*β* (product no.: SC-7149), and rabbit polyclonal antibody against phosphorylated-p38 (product no.: SC-101759) were purchased from the Santa Cruz Corporation, USA. A rabbit polyclonal antibody against ATF-2 (product no.: AB11031) was purchased from the Abcam Company. HRP-labeled goat anti-rabbit IgG (product no.: ZDR-5306) was obtained from the ZSGB-Bio Corporation, and Protein-marker was obtained from the MBI Company. The developer and fixative solutions were purchased from Applygen Company (Beijing).

### 2.2. Endothelial Cell Culture and the Establishment of the Ischemia-Reperfusion Injury Model

#### 2.2.1. Endothelial Cell Culture

A frozen aliquot of cells was obtained from liquid nitrogen and immediately thawed at 37°C. The cells were injected into a polylysine (20 mg/L)-coated culture bottle and cultivated in an incubator with 95% O_2_ and 5% CO_2_ at 37°C. The cells were passaged every 2 to 3 days, and the endothelial cells were used in the following experiments at passage 4 or 5.

#### 2.2.2. Establishment of the Ischemia-Reperfusion Injury Model

Endothelial cells at 80% confluency were digested into single cell suspension, and the cell density was adjusted to 1 × 10^5^/mL. The cells were subsequently seeded onto polylysine-coated 96-well plates. A volume of 100 *μ*L cell suspension was added to each well, and the media was replaced the next day. The IRI model was established at one day later. Ischemia simulation: the cells were washed twice with PBS, changed into sugar-free Earle's solution, and subsequently cultivated in an incubator with 1% O_2_ and 5% CO_2_ at 37°C. Reperfusion simulation: the sugar-free Earle's solution was replaced with complete medium, and the cells were further cultivated in a CO_2_ incubator.

#### 2.2.3. Evaluation of the IRI Model

(1) *Evaluation of the Endothelial Cell Viability*. The MTT method was used to assess the cell viability. Four hours before the end of the ischemic reperfusion, 20 *μ*L of MTT solution was added to each well. Subsequently, the supernatant was discarded prior to the addition of 150 *μ*L DMSO, and the cells were gently oscillated for 10 minutes. The cell viability was detected using an enzyme-labeling instrument at 492 nm. The hypoxia duration was 1, 2, and 4 hours, while reoxygenation lasted 6 and 24 hours.

(2) *Rate of Adhesion between HCMECs and PMN*. Preparation of PMN: elbow vein blood was collected from healthy adults into tubes containing heparin anticoagulant and mixed well with an equal volume PBS. The samples were placed onto the liquid surface of PMN separation medium. The mixture was centrifuged at 2000 r/min for 20 minutes. The third layer of PMN was collected and washed 3 times with PBS. The ECM was used to resuspend the PMN, and the cell density was adjusted to 1 × 10^9^/mL. Subsequently, 0.05 mL of the cell suspension was mixed with an equal volume of trypan blue (10 g/L) for staining, showing 95% PMN viability. Moreover, Giemsa staining showed that the purity of the PMN cell suspension was more than 97%. The cell suspension was stored at 4°C for subsequent use within 2 hours.

HCMECs and PMN adhesion experiment: endothelial cells were treated using the hypoxia/reoxygenation method described above, and the ECM was removed from the 96-well, followed by the addition of 200 *μ*L of PMN cell suspension (1 × 10^9^/L). Subsequently, the cells were incubated in a CO_2_ incubator for 1 h. The unadhered PMN were discarded through aspiration, and the remaining cells were washed twice with PBS. A total of 100 *μ*L of Rose Bengal sodium salt (2.5 g/L) was added to each well, and the reaction was incubated at room temperature for 10 minutes. The dyeing solution was subsequently discarded, and the cells were washed twice with PBS. The cells were mixed with an equal proportion of PBS (0.1 g/L) and 0.95% ethanol for decolourisation. Subsequently, the cells were stored at room temperature for 1 h, and adhesion was detected using an enzyme-labeling instrument at 550 nm. The hypoxia duration was 2 h, and reoxygenation lasted 6 and 24 h.

### 2.3. Effects of Astragalus Polysaccharide on the Adhesion of HCMECs and PMN during IRI

#### 2.3.1. The Experiment Comprised Six Groups

Control group (cells were cultured normally without stimulation); hypoxia/reoxygenation group (2-hour oxygen and glucose deprivation, followed by 24-hour reoxygenation and resupply of glucose); treatment with low, moderate, and high doses of Astragalus Polysaccharide (25, 50, and 100 mg/L of astragalus polysaccharide, respectively, were added during hypoxia/reoxygenation), SB203580 group (50 *μ*moL/L SB203580 was added for 30 minutes prior to hypoxia and subsequently supplanted with sugar-free Earl's solution using the same stimulating method as performed in the hypoxia/reoxygenation group). Six replicates were performed in separate wells for each group.

#### 2.3.2. Rate of Adhesion between HCMECs and PMN

After 2 hours of hypoxia treatment, followed by up to 4 hours of reoxygenation, the rate of adhesion between HCMECs and PMN was determined using the methods described above.

### 2.4. Influence of Astragalus Polysaccharide on Adhesion Molecules (P-Selectin and E-Selectin) and Gene Transcription through the p38 MAPK Signalling Pathway (p38, ATF-2) in HCMECs under IRI

The groups were assigned in the same manner as described above. The mRNA was extracted, reverse transcribed, and quantified using real-time PCR. The following PCR primers were used:

E-selectin (238 bp): Forward 5′-GCA CAT CTC AGG GAC AAT GGA-3′, Reverse 5′-TTG GAC TCA GTG GGA GCT TCA-3′;

P-selectin (306 bp): Forward 5′-TTC AGG ACA ATG GAC AGC AGT-3′, Reverse 5′-GTC CCA CCC ATT ATC AGA CCT-3′;

p38 (260 bp): Forward 5′-GCC GAA GAT GAA CTT TGC GA-3′, Reverse 5′-GTG GTG GCA CAA AGC TGA TG-3′;

ATF-2 (237 bp): Forward 5′-ATG GTC AGC TGC AGA GTG AAG-3′, Reverse 5′-CTG CCT TGG AGG TTG AAC TGA-3′;

*β*-actin (302 bp): Forward 5′-TCC TCC CTG GAG AAG AGC TA-3′, Reverse 5′-TCA GGA GGA GCA ATG ATC TTG-3′.

The reaction conditions included predenaturation at 95°C for 10 min, followed by 40 cycles of denaturation at 95°C for 30 s, renaturation at 55°C for 30 s, and elongation at 72°C for 30 s. The Stratagene MX3000P System was used to analyse the data and compare the P-selectin and E-selectin mRNA ratios among the different groups.

### 2.5. Influence of Astragalus Polysaccharide on Adhesion Molecules (P-Selectin and E-Selectin) and Protein Expression through the p38 MAPK Signalling Pathway (p38/Phosphate-p38, ATF-2) in HCMECs under IRI

The cells were lysed in cell lysis buffer (15 mM Tris-HCl, pH 7.5, 0.2% TritonX-100, and 150 mM NaCl), and the cellular protein extract was collected. The proteins were separated using 10% SDS-PAGE and transferred to nitrocellulose membranes, which were subsequently incubated with primary antibody (1/1000) at 4°C. The membranes were further incubated with horseradish peroxidase-conjugated secondary antibody (goat anti-rabbit) (1 : 2000) for 2 h at room temperature, followed by ECL visualisation. A gel imaging system (Syngene Co.) was used to capture the images, which were subsequently analysed using Image J software (NIH image, Bethesda, MD).

### 2.6. Statistical Analyses

The data were expressed as the means ± SEM. The statistical evaluation was performed using SPSS10.0 software. The statistical comparisons were performed using a one-way analysis of variance (ANOVA), and Dunn's method was used to discriminate the differences between different groups. *P* < 0.05 was considered statistically significant.

## 3. Results

### 3.1. Establishment of the Ischemia-Reperfusion Model *In Vitro*

#### 3.1.1. Endothelial Cell Culture

**Figure 1:**
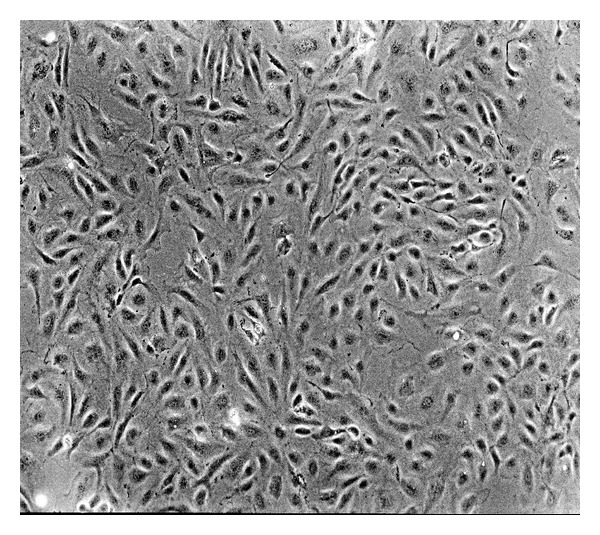
HCMECs cultivated for 3 days (100x).

HEMECs were grown into a confluent single layer; the cell body was plump and transparent, with a rhombic or polygonal shape and a small round nucleus (Figure 1).

#### 3.1.2. Viability of Endothelial Cell after Ischemia-Reperfusion

**Figure 2:**
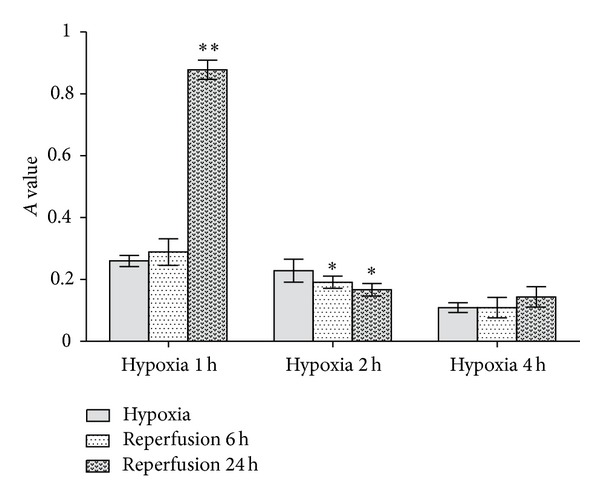
Viability of endothelial cells after ischemia-reperfusion. In contrast to 1 h oxygen deprivation alone, 1 h followed by 6 h reperfusion will not cause further injury to HCMEC; however, 24 h reperfusion would lead to apparent IRI to HCMEC. Compared with 2 h hypoxia alone, 2 h hypoxia followed by 6 h reperfusion or 12 h reperfusion both can result in IRI. In comparison with 4 h oxygen deprivation alone, 4 h hypoxia followed by 4 h reperfusion, 6 h reperfusion, or 24 h reperfusion all bring about similar influence on cell proliferation. (*P < 0.05, **P < 0.01 versus the corresponding hypoxia-treated groups.)

The cell viability remained unchanged after 1 h hypoxia, followed by 6 h reperfusion. When the reperfusion duration was prolonged to 24 h, the cell activity increased dramatically compared with the 1 h hypoxia group (*P* = 0.000). The comparison between the 2 h hypoxia group and the 2 h hypoxia, followed by 6 and 24 h reperfusion groups, revealed that the viability of latter group was markedly lower (*P* = 0.041, 0.035). However, after 4 h of hypoxia, followed by 6 and 24 h of reperfusion, the cell activity was similar to that of cells subjected to 4 h hypoxia treatment alone (Figure 2).

#### 3.1.3. Rate of Adhesion between Endothelial Cells and PMN

**Figure 3:**
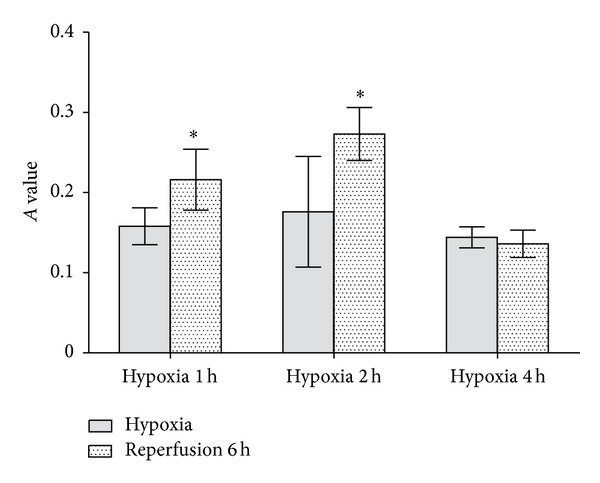
Rate of adhesion between endothelial cells and PMN. In contrast to 1 h oxygen deprivation alone, 1 h hypoxia followed by 6 h reperfusion increases the number of neutrophil granulocytes that adhered to HCMEC. Same result happens to comparison between 2 h hypoxia group and 2 h hypoxia followed by 6 h reperfusion group. Which means more neutrophil granulocyte adhere to HCMEC in latter group than in former group. However, things become different in 4 h hypoxia group and 4 h oxygen deprivation followed by 6 h reperfusion group. That is to say cohesion between neutrophil granulocytes and HCMEC in two groups is of no significant difference. (*P < 0.05 versus the corresponding hypoxia-treated groups.)

The adhesion rates after 1 h hypoxia, followed by 6 h reperfusion and 2 h hypoxia, followed by 6 h reperfusion, were significantly different from the corresponding groups were subjected to hypoxia alone (*P* = 0.030, 0.010). Both groups of cells subjected to 4 h hypoxia, and 4 h hypoxia, followed by 4 h reperfusion exhibited low adhesion rates and there was no difference between them (*P* = 0.803) (Figure 3).

### 3.2. Effects of Astragalus Polysaccharide on Adhesion between PMN and HCMECs Subjected to IRI

**Figure 4:**
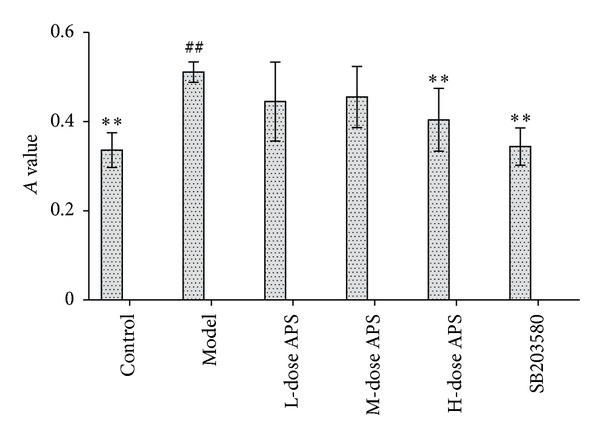
Effects of astragalus polysaccharide on the adhesion between PMN and HCMECs subjected to IRI. The number of attached cells after treatment with high-dose astragalus polysaccharide and SB203580 was markedly different from the number of cells observed in the model group. (^##^ *P* < 0.01 versus the control group. **P < 0.01 versus the model group.)

The adhesion of the cells in the reperfusion groups was obviously higher than that observed in the control group (*P* = 0.001). Treatment with astragalus polysaccharide decreased HCMECs-PMN adhesion. Specifically, the number of attached cells after treatment with high-dose astragalus polysaccharide and SB203580 was markedly different from the number of cells observed in the model group (*P* = 0.011, 0.000) (Figure 4).

### 3.3. Influence of Astragalus Polysaccharide on the Gene Transcription of Adhesion Molecules (P-Selectin and E-Selectin) and p38 Signalling Pathway Factors in HCMECs during IRI

**Figure 5:**
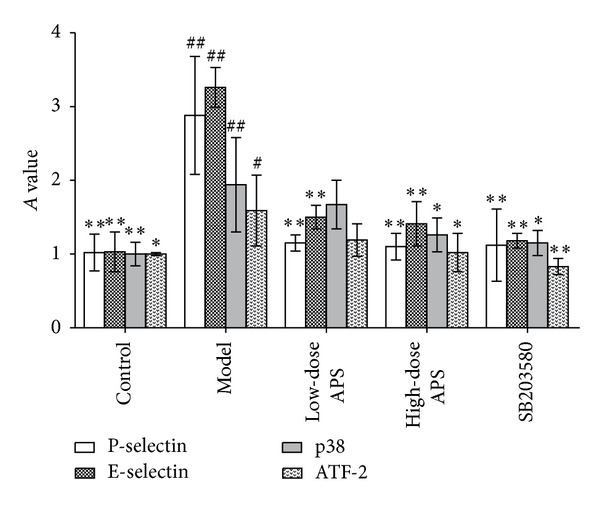
Gene transcription of adhesion molecules and p38 signalling pathway-related proteins in HCMECs during IRI. (^#^ *P* < 0.05, ^##^ *P* < 0.01 versus the control group. *P < 0.05, **P < 0.01versus the model group.)

The results demonstrated that the gene transcription of P-selectin, E-selectin, p38, and ATF-2 in the model group was higher than in the control group (*P* = 0.000). In the low- and high-dose astragalus polysaccharide treatment groups and the SB203580 group, the transcription of P-selectin and E-selectin was significantly inhibited compared with the model group (*P* = 0.000). In addition, high-dose astragalus polysaccharide and SB203580 treatments suppressed the transcription of p38 and ATF-2 during IRI (Figure 5). 

### 3.4. Influence of Astragalus Polysaccharide on the Protein Expression of Adhesion Molecules (P-Selectin and E-Selectin) and p38 Signalling Pathway Factors (p38, p-p38, and ATF-2) in HCMECs during IRI

**Figure 6:**
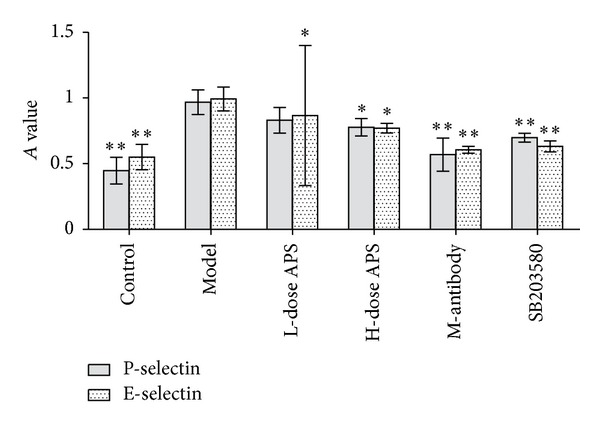
Protein expression of adhesion molecules. High-dose astragalus polysaccharide and SB203580 treatments decreased P-selectin and E-selectin expression (*P < 0.05, **P < 0.01 versus the model group.)

**Figure 7:**
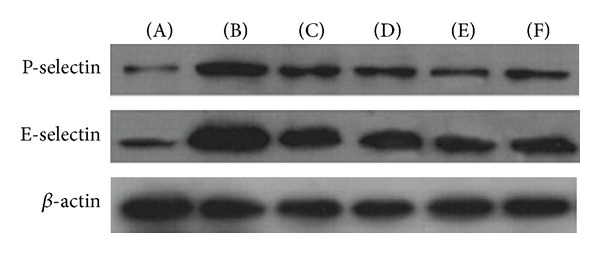
Protein expression of adhesion molecules. (A) Normal control group, (B) model group, (C) low-dose AP group, (D) high-dose AP group, (E) cohesion molecule antibody group, and (F) SB203580 group.

**Figure 8:**
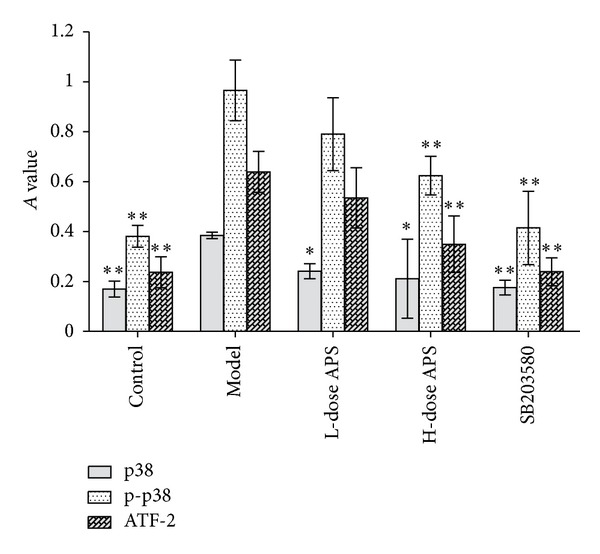
Protein expression of the factors involved in p38 MAPK signalling pathways. High-dose astragalus polysaccharide and SB203580 treatments decreased p38, p-p38 and ATF-2 expression (*P < 0.05, **P < 0.01 versus the model group.)

**Figure 9:**
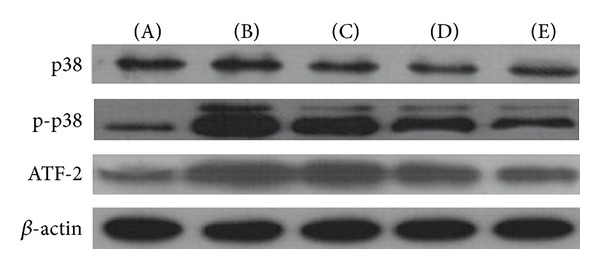
Protein expression of the factors involved in p38 MAPK signalling pathways. (A) Normal control group, (B) model group, (C) low-dose AP group, (D) high-dose AP group, and (E) SB203580 group.

Compared with the control group, the expression of the adhesion molecules (P-selectin and E-selectin) and p38 signalling pathway factors (p38, p-p38 and ATF-2) was significantly elevated (*P* < 0.01. *P* = 000, 0.000, 0.006, 0.000, and 0.000). High-dose treatments with astragalus polysaccharide, SB203580, and adhesion molecule antibodies suppressed the effects on the expression of the 5 proteins described above in HCMECs during IRI (*P* < 0.01. P-selectin = 0.133, 0.044, 0.007, 0.001. E-selectin = 0.031, 0.017, 0.001, 0.000. p38 = 0.041, 0.018, 0.007. p-p38 = 0.090, 0.004, 0.000. ATF-2 = 0.190, 0.003, 0.000) (Figures 6, 7, 8, and 9).

## 4. Discussion

IRI is a type of inflammation reaction mediated through PMN. It has been suggested that this mechanism involves activated leukocytes that adhere to the vascular endothelium and obstruct capillaries, thereby decreasing the blood flow and compromising the integrity and function of the capillaries [15, 16]. Apart from the direct damage to endothelial cells, PMN also migrate to ischemic cardiac muscles and release large amount of active substances, leading to elevated vascular permeability, tissue edema, tissue necrosis and cardiac myocyte apoptosis [17, 18]. It is clear that the inhibition of adhesion between PMN and endothelial cells alleviates IRI. In the present study, we showed that astragalus polysaccharide possesses anti-IRI and anti-PMN adhesion effects using an IRI model *in vitro*, consistent with those of the results obtained in our previous animal experiments. Thus, this compound, derived from the herb blood supply, relieves cardiac muscle damage in rats subjected to IRI.

When PMN adhere to the endothelium of coronary arteries after IRI, the molecules mediating adhesion might be the initial factors facilitating cardiac muscle IRI. Under normal conditions, PMN and HCMECs seldom adhere, and occasional adherence is rapidly dissociated [19]. In contrast, after IRI, endothelial cells and adhesion molecules from the PMN surface vary in number and structure, contributing to the substantial attachment of the PMN to the endothelium surface. Unfortunately, the mechanism underlying this phenomena is not yet fully understood. Recent studies have indicated that hypoxia/reoxygenation stimulates endothelial cells to release active substances, such as oxygen radicals, which induce expression and formation changes in adhesion molecules from PMN and activate adhesion molecules on the surface of PMN, thereby potentiating cell adhesion.

In the present study, we demonstrated that astragalus polysaccharide possesses restraining effects on the transcription and expression of two adhesion molecules, P-selectin and E-selectin. P-selectin is expressed in the Weibel-Palade body of endothelial cells and is secreted from the cell interior to the serous membrane on the cell surface minutes after reperfusion occurs, to perform its PMN-recruiting function [20]. Thereafter, P-selectin is degraded into soluble fragments [21]. Hillis and coworkers suggested that elevated soluble P-selectin in blood might serve as an indicator to predict early-stage myocardial ischemia in chest pain patients [22]. In contrast, E-selectin is not expressed under normal conditions but rather is secreted during endothelial cell stimulation. However, the synthesis of E-selectin is slow and is typically initiated at 4 to 6 hours after hypoxia [23]. Thus, E-selectin is not an important factor during the early hours after IRI; instead, this factor plays a vital role in unconsolidated PMN adhesion during the later stages of IRI [24]. Astragalus polysaccharide reduces P-selectin and E-selectin expression during early- and late-stage IRI, respectively. Thus, astragalus polysaccharide affects the entire course of PMN-endotheliocyte adhesion, and the differences in stimulating methods and cells utilised in this experiment might reflect the inconsistencies between our results and those of others [13]. HCMECs were used in this study, as these cells represent the first battle line in IRI; that is, these cells are the first affected sites in reperfusion. Therefore, changes in HCMECs exert huge influences on the damage or restoration of cardiac muscle [25, 26]. In addition, recent studies have shown that endothelial cells of different organic and histological origins obviously vary in morphology, gene, and function. Consequently, the phenomenon that large vessels and microvessels are derived from endothelial cells in animal deserves further reflection [27–29]. Thus, to avoid the problems described above, we used HCMECs in this study to yield more realistic results. 

Based on the fact that the p38 signalling pathway is one of the major channels regulating inflammation reactions, we proposed that astragalus polysaccharide likely regulates this pathway. In the present study, astragalus polysaccharide, isolated from *Scutellariae baicalensis *Georgi, is effective in suppressing p38 phosphorylation and inhibiting the expression of ATF-2, a downstream p38 effector molecule; consequently, the modulatory activity of astragalus polysaccharide affects the transcription and protein synthesis of cohesion molecules. 

In a previous animal study, we showed that astragalus polysaccharide exerts a positive effect on the management of myocardial ischemia. Based on the results obtained in the present study, the adhesion molecules in HCMECs are the central targets for the anti-IRI effects of astragalus polysaccharide.

## 5. Conclusions

The results of this study suggest that treatment with astragalus polysaccharide, within a certain dose range, suppresses the adhesion between HCMECs and PMN, and the underlying mechanism of this suppression is associated with the downregulation of the expression and phosphorylation of p38 MAPK, thereby contributing to the subsequent expression of P-selectin and E-selectin. These results are consistent with the findings obtained in previous animal experiments demonstrating that this compound possesses anti-IRI effects. The qi-reinforcing and yang-elevating effects of astragalus polysaccharide treatment for IRI in traditional Chinese medicine can therefore be interpreted as the regulation of the expression of major cohesion molecules in HCMECs and the revascularisation of microcirculation.
